# The Functional Role of Voltage-Gated Sodium Channel Nav1.5 in Metastatic Breast Cancer

**DOI:** 10.3389/fphar.2020.01111

**Published:** 2020-07-23

**Authors:** Qianxuan Luo, Ting Wu, Wenfang Wu, Gong Chen, Xuan Luo, Liping Jiang, Huai Tao, Mingqiang Rong, Shuntong Kang, Meichun Deng

**Affiliations:** ^1^Department of Biochemistry and Molecular Biology & Hunan Province Key Laboratory of Basic and Applied Hematology, School of Life Sciences, Central South University, Changsha, China; ^2^Hunan Key Laboratory of Animal Models for Human Diseases & Hunan Key Laboratory of Medical Genetics, School of Life Sciences, Central South University, Changsha, China; ^3^Xiangya School of Medicine, Central South University, Changsha, China; ^4^Department of Biochemistry and Molecular Biology, Hunan Normal University, Changsha, China; ^5^Department of Biochemistry and Molecular Biology, Hunan University of Chinese Medicine, Changsha, China

**Keywords:** VGSCs, Nav1.5, breast cancer, metastasis, mechanism, inhibitors

## Abstract

Voltage-gated sodium channels (VGSCs), which are abnormally expressed in various types of cancers such as breast cancer, prostate cancer, lung cancer, and cervical cancer, are involved in the metastatic process of invasion and migration. Nav1.5 is a pore-forming α subunit of VGSC encoded by *SCN5A*. Various studies have demonstrated that Nav1.5, often as its neonatal splice form, is highly expressed in metastatic breast cancer cells. Abnormal activation and expression of Nav1.5 trigger a variety of cellular mechanisms, including changing H^+^ efflux, promoting epithelial-to-mesenchymal transition (EMT) and the expression of cysteine cathepsin, to potentiate the metastasis and invasiveness of breast cancer cells *in vitro* and *in vivo*. Here, we systematically review the latest available data on the pro-metastatic effect of Nav1.5 and its underlying mechanisms in breast cancer. We summarize the factors affecting Nav1.5 expression in breast cancer cells, and discuss the potential of Nav1.5 blockers serving as candidates for breast cancer treatment.

## Introduction

Breast cancer accounts for the largest proportion of women’s mortality worldwide (Greaney et al., 2015). In the United States, approximately 12% of women will develop invasive breast cancer during their lifetime. The diagnosis of at least 276,480 new cases of invasive breast cancer and 42,170 breast cancer associated deaths are estimated in 2020 (Breastcancer.org). It is a complicated disease with inter- and intra-tumoral heterogeneity, and there are several different classifications of breast cancer according to advanced technical methods, including histological stratification and gene expression clustering (Sorlie et al., 2001; Margan et al., 2016; Yeo and Guan, 2017). The different molecular types of breast cancer correspond to different molecular mechanisms and require specific therapeutic strategies (Prat et al., 2015; Arciero et al., 2019). Therefore, appropriate management and therapies are necessary to achieve the best response to improve the prognosis and survival of patients. Notably, breast cancer cells have the potential to metastasize to distant organs such as the brain, lung, liver, and bone (Hoshino et al., 2015; Hurvitz et al., 2018; Park et al., 2018; Zhu et al., 2019). Cancer metastasis is a complex, multi-step process that is closely associated with cells’ local invasion, blood and lymphatic diffusion, and extravasation and colonization at distant sites. Metastatic cancer cells degrade the extracellular matrix, detach from their original location, and enter the circulation system through which they reach and colonize distant organs (Mego et al., 2010; Massague et al., 2017). Metastasis is the direct cause of death from cancer, accounting for nearly 90% of deaths related to breast cancer (Xie et al., 2017). Thus, there is an urgent need to identify the underlying molecular mechanisms of breast cancer and develop effective therapeutic strategies.

Voltage-gated sodium channels (VGSCs) are primarily expressed in excitable cells such as neurons. Na^+^ influx regulated by VGSCs produces action potential and propagates excitability. VGSCs also exist in non-excitable cells, including microglia, astrocytes, immune cells, fibroblasts, and cancer cells, where they regulate and influence an array of biological functions such as phagocytosis, motility, Na^+^/K^+^-ATPase activity, and metastatic activity (Black and Waxman, 2013). VGSCs are composed of pore-forming α subunits and auxiliary β subunits (Hull and Isom, 2018). The family Nav1 comprises nine genes (*SCN1A*-*5A*, *SCN8A*-*11A*) that encode α subunits, Nav1.1 to Nav1.9. Each α subunit comprises four homologous domains, and each domain consists of six transmembrane segments (Catterall, 2000). Five β subunits, including β1 and its splice variants β1B and β2–β4, are encoded by four genes (*SCN1B*-*SCN4B*) (Brackenbury and Isom, 2011). These β subunits have been found to modulate the bioelectrical characteristics of α subunits and function as cellular adhesion molecules. Both α and β subunits play a critical role in the development of the central nervous system (CNS), and altering the expression of specific subunits may cause developmental aberrations and CNS lesions. Furthermore, expression of α and β subunits is upregulated in various cancers such as breast cancer, prostate cancer, lung cancer, and cervical cancer, observed both in *in vitro* and *in vivo* systems. They are found to enhance metastasis progression, including invasion, migration, endocytosis, and gene expression, *via* the Na^+^ currents carried by α subunits and the adhesion interaction regulated by β subunits (Yang et al., 2012). The correlation between VGSCs-inhibiting drug uses and outcomes of cancer patients has been highlighted recently (Fairhurst et al., 2015; Reddy et al., 2015; Kao et al., 2018), even though the conclusions are still inconsistent. Hence, randomized controlled trials are required to exclude confounding factors and obtain a convincing conclusion in the future.

Nav1.5, a pore-forming α subunit of VGSC encoded by *SCN5A*, is expressed in lymphoma, neuroblastoma, breast and prostate cancer cells. Aberrant expression and activity of Nav1.5 are associated with cardiac excitability diseases such as arrhythmic dilated cardiomyopathy, Brugada syndrome, and long QT syndrome (Antzelevitch et al., 2005; Amin et al., 2013; Giudicessi and Ackerman, 2013; Beckermann et al., 2014). Nav1.5 is also overexpressed in metastatic breast cancer *in vitro* and *in vivo* and mainly exists in its DI: S3 5′ neonatal splice form (nNav1.5) in breast cancer cells, potentiating cell metastasis (Mohammed et al., 2016; Yamaci et al., 2017). Breast cancer cells treated with specific Nav1.5 inhibitors or siRNAs show decreased motility and metastatic capacity (Driffort et al., 2014).Therefore, Nav1.5 may be regarded as a promising target for the diagnosis and therapy of breast cancer.

With the high motility and metastatic capacity of breast cancer, it is imperative to determine the mechanisms of pro-metastatic effects of Nav1.5 and develop effective Nav1.5 inhibitors for breast cancer treatment. This review clarifies the role and mechanisms of Nav1.5 in metastatic breast cancer progression and summarizes some drugs with remarkable effects on reducing metastasis of breast cancer by acting on Nav1.5. All these evidence supports the idea that Nav1.5 as an anti-metastatic target for the treatment of metastatic breast cancer.

### Nav1.5 Expression and Its Functional Role in Breast Cancer Metastasis

Nav1.5 in its neonatal DI:S3 5′ splice form is predominantly expressed in metastatic cancer cells (Fraser et al., 2005; Yamaci et al., 2017). This form has been found to participate in neonatal development, while it is absent in postnatal development. The overexpression of Nav1.5 in cancer cells suggests that embryonic genes are re-expressed during ontogenesis and participate in many cellular behaviors related to metastasis (Monk and Holding, 2001).

The expression levels of Nav1.5 and nNav1.5 in the highly metastatic MDA-MB-231 breast cancer cell line were significantly higher than those in weakly metastatic MCF-7 cells (Kamarulzaman et al., 2017; Zhang et al., 2018). Nav1.5 is specifically present on the membrane of MDA-MB-231 cells, but not in normal cell lines and weakly metastatic MCF-7 cells. In breast cancers, Nav1.5 α subunit mRNA and protein expression correlates with metastatic potential, and the neonatal *SCN5A* splice variant is expressed ~1,800-fold higher in metastatic MDA-MB-231 cells than in weakly metastatic MCF-7 cells. When voltage-gated membrane currents are examined in different cell lines, inward currents only occur in the highly metastatic breast cancer cell line MDA-MB-231 (Fraser et al., 2005). *In vivo*, it is widely acknowledged that Nav1.5 is present in breast cancer biopsies and is related to lymph node metastasis (Fraser et al., 2005). *SCN5A* expression is significantly elevated in breast cancer tissues and is an independent predictor of poor prognosis compared to its expression in normal breast tissue. *SCN5A* is overexpressed in tumor samples from patients who experience recurrence and death within 5 years; thus, *SCN5A* overexpression is associated with increased odds of developing metastasis (Yang et al., 2012). Nelson and his colleagues investigated the functional activity of Nav1.5 and its specific contribution to breast cancer tumor progression. *SCN5A* is upregulated at the mRNA and protein levels in metastatic breast tumors compared to that in normal, non-cancerous tissue (Nelson et al., 2015a; Nelson et al., 2015b; Yamaci et al., 2017)

Furthermore, some factors affect Nav1.5 expression in breast cancer cells. The β1 subunit mRNA and protein are strongly expressed in MCF-7 cells and are barely detectable in MDA-MB-231 cells. Inhibition of the β1 subunit reduces adhesion and enhances metastatic cell behavior by upregulating nNav1.5 expression (Chioni et al., 2009). It has been reported that the expression level of repressor element silencing transcription factor (REST) is significantly lower in MDA-MB-231 cells than in MCF-7 cells (Kamarulzaman et al., 2017). The inhibition of REST results in re-expression of various neonatal genes (Kuwahara, 2013), and REST recruits histone deacetylases (HDACs) for transcriptional repression activity (Roopra et al., 2000). It has been postulated that downregulation of REST and HDAC2 expression levels enhances the expression of Nav1.5 and nNav1.5, promoting aggressiveness of tumors (Kamarulzaman et al., 2017). The sigma-1 receptor is known as a protein located on the plasma membrane, endoplasmic reticulum, and perinuclear areas (Hayashi and Su, 2003; Hayashi and Su, 2005), and is up-regulated in breast cancer cells and tissues (Aydar et al., 2016). In breast cancer cells, the sigma-1 receptor combines with nNav1.5 protein to form a complex with a four-fold symmetry (Balasuriya et al., 2012), which translocate nNav1.5 protein to the plasma membrane, thereby increases the metastatic activity (Aydar et al., 2016). Salt-inducible kinase 1 (SIK1) has been identified as an important factor in regulating sodium homeostasis and as a tumor repressor that participates in the progression of cancer cells (Sjostrom et al., 2007; Selvik et al., 2014). Lower expression of SIK1 was observed in different breast tumor grades than in normal tissues (Gradek et al., 2019). Silencing SIK1 upregulates *SCN5A* expression and increases H^+^ outflow, thus, improving invasiveness (Gradek et al., 2019). TGF-β1 is a well-known inducer of epithelial-to-mesenchymal transition (EMT) and a multifunctional regulatory factor affecting cancer development (Jakowlew, 2006; Moustakas and Heldin, 2016). Weakly metastatic MCF-7 cells treated with TGF-β1 demonstrated increased expression of *SCN5A* and induction of invasion (Gradek et al., 2019). Epidermal growth factor (EGF) signals have been confirmed to be overexpressed and are involved in the development of breast cancer (Adams et al., 2009; Yao et al., 2012). Treatment with EGF promoted the migratory capacity of MDA-MB-231 cells by increasing the functional expression of the Nav1.5 channel (Gonzalez-Gonzalez et al., 2019). These results confirm that Nav1.5 channels play an important role in human breast cancer by affecting metastatic activity.

Furthermore, the importance of Nav1.5 expression in human breast cancer cells for the colonization of organs was assessed. ShCTL or shNav1.5 breast cancer cells injected into mice. ShCTL cells expressing Nav1.5 strongly colonized and invaded the chest area. All mice showed lung colonization and some showed rachis and rib colonization (Driffort et al., 2014). In addition, a high level of nNav1.5 expression is associated with the estrogen receptor (ERα) status of breast cancer. In all cases, lack of ERα was positive for nNav1.5 expression. In all cases of negative nNav1.5 expression, ERα is present (Yamaci et al., 2017). It is widely acknowledged that breast cancer cases lacking ERα protein are closely related to a more advanced stage and have a worse prognosis (Yi et al., 2014; Zhou and Slingerland, 2014). This is consistent with previous results showing that a high level of nNav1.5 expression is associated with aggressive breast cancer development. Consequently, nNav1.5 expression enhances growth and metastatic dissemination of breast cancer and is a potential prognostic marker for breast cancer.

### Mechanisms of Action

Metastasis of breast cancer is a complex process, and there are a considerable number of studies focusing on this topic. Nav1.5 is involved in the metastatic cascade in breast cancer by acting on different targets.

**Figure 1:**
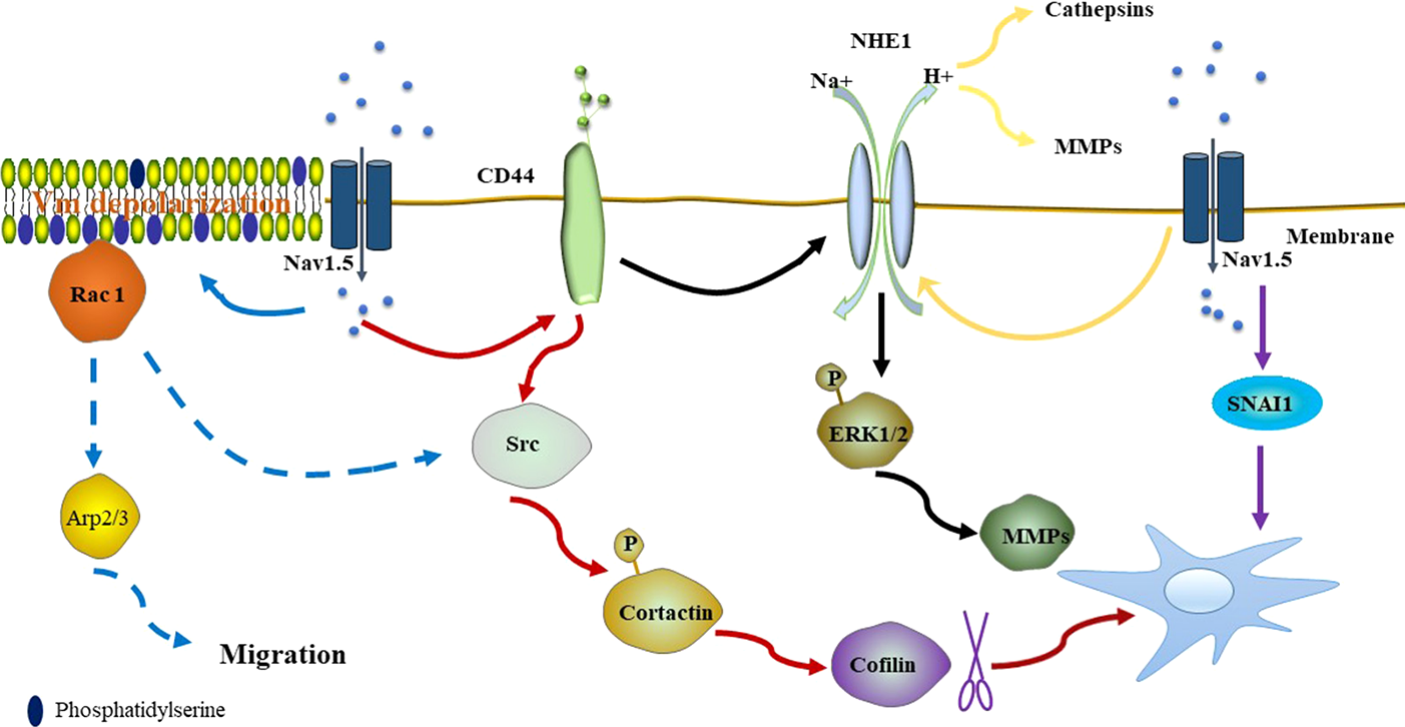
Nav1.5 promotes the activity of NHE-1, resulting in the acidification of the peri-membrane, which favors the proteolytic activity of Cat and MMPs **(yellow line)**. Nav1.5 also promotes invasiveness by the CD44-src-cortactin signaling pathway. After phosphorylation, cortactin releases actin-severing cofilin, promoting the formation of actin barbed ends of invadopodia **(red line)**. Overexpression of CD44 upregulates the expression and activity of NHE-1, resulting in the expression level of MMPs (MMP2, MMP9, and MMP14) by increasing phosphorylated ERK1/2 **(black line)**. Nav1.5 dependent Vm depolarization regulates Rac1 activation by its interaction with phosphatidylserine **(blue solid line)**. Rac1 regulates the Arp2/3 complex and is closely associated with phosphorylation of cortactin and cofilin, promoting the promigratory phenotype **(blue dotted line)**. High expression of Na_V_1.5 induces expression of SNAI1, triggering the EMT and pro-metastatic effect of breast cancer cells **(purple line)**. (Dotted line indicates that the mechanism exists but has not been confirmed in breast cancer).

Na^+^ currents carried by Nav1.5 have been found to promote the invasiveness of the breast cancer cell line MDA-MB-231 by regulating the H^+^ influx carried through Na^+^/H^+^ exchanger isoform-1 (NHE-1). NHE-1, which is an important regulator of H^+^ efflux, is co-expressed with Nav1.5 in lipid rafts within the caveolae of MDA-MB-231 cells. NHE-1 and Nav1.5 are coupled in the metastatic process of MDA-MB-231 cells (Brisson et al., 2011). Na^+^ inward currents can be found in highly metastatic MDA-MB-231 cells but are absent in weakly metastatic or normal cells. Similarly, highly metastatic MDA-MB-231 cells express a high level of NHE-1 mRNA, while weakly metastatic MCF-7 cells express a low level of NHE-1 mRNA, and normal cells express no NHE-1 (Chen et al., 2019). In MDA-MB-231 cells, Nav1.5 allosterically modulates the activity of NHE-1, rendering NHE-1 more active at physiological intracellular pH. This results in increased H^+^ efflux to the extracellular space, leading to acidification of the peri-membrane and intracellular alkalization (Brisson et al., 2013; Amith and Fliegel, 2017). The environment of the perimembrane favors the proteolytic activity of cysteine cathepsins (Cat) and matrix metalloproteinases (MMPs), which are major drivers of extracellular matrix (ECM) degradation and increase the invasiveness of breast cancer cells (**Figure 1**). Cat C, Cat B, Cat K, and Cat L are involved in Cat-dependent invasiveness in MDA-MB-231 cells. Apart from Cat K, the activity of this proteolytic protein can be potentiated by Nav1.5 (Gillet et al., 2009; Robey and Nesbit, 2013). NHE-1–modulated invasiveness can also be found in hepatoma cells (Yang et al., 2011).

Nav1.5 expression enhances src kinase activity and controls cortactin phosphorylation, resulting in the release of actin-severing cofilin and the formation of actin barbed ends of invadopodias, which are actin-rich organelles that enable cells to stretch into the ECM and perform their proteolytic effect (Oser et al., 2009; Brisson et al., 2013). It is known that Nav1.5 expression in breast cancer cells is closely associated with the protein level of CD44, which correlates with poor outcomes of breast cancer (McFarlane et al., 2015; Nelson et al., 2015b). In turn, CD44 adheres to its ligand and leads to the activation of src and phosphorylation of cortactin (Bourguignon et al., 2001; Hill et al., 2006), thus, supporting that Nav1.5 may potentiate the invasion of breast cancer cells by the CD44-src-cortactin signaling axis. Apart from the CD44-src-cortactin signaling axis, CD44 targets NHE-1 to regulate the metastatic capacities of MDA-MB-231 cells through the mitogen-activated protein kinase pathway. Overexpression of CD44 upregulates the expression and activity of NHE-1, resulting in the increased expression of MMPs (MMP2, MMP9, and MMP14) by increasing phosphorylated ERK1/2 (**Figure 1**). Moreover, CD44 accelerates tumor growth and metastasis to the lung in MDA-MB-231 cells (Chang et al., 2014).

Increasing evidence shows that plasma membrane potential (Vm) is related to cell cycle progression and the level of differentiation, development, regeneration, and migration of cancer cells (Sundelacruz et al., 2009; Yang and Brackenbury, 2013). Emerging evidence confirms that proliferating tumor cells depolarizes Vm, while terminally differentiated non-cancer cells are characterized by hyperpolarized Vm (Binggeli and Cameron, 1980; Marino et al., 1994; Fraser et al., 2005). Yang et al. reported that a steady-state inward Na^+^ current carried by Nav1.5 contributes to depolarizing the resting Vm in MDA-MB-231 cells (Yang et al., 2020). Previous studies suggest that Vm depolarization promotes the redistribution of anionic phospholipids PIP_2_ and phosphatidylserine within the plasma membrane, resulting in GTPase K-Ras activation (Chen et al., 2011; Zhou et al., 2015; Remorino et al., 2017). Rac1 is one of the Rho GTPases that contribute to lamellipodia formation and migration (Nobes and Hall, 1995; Steffen et al., 2013). Similarly, Nav1.5 dependent Vm depolarization regulates Rac1 activation and localization in lamellipodia by its interaction with phosphatidylserine, regulating cell morphology and migration in breast cancer cells (Yang et al., 2020). Rac1 is a potential upstream regulator of the Arp2/3 complex, which is required for lamellipodia extension (Suraneni et al., 2012). Furthermore, Rac1 is closely associated with phosphorylation of cortactin and cofilin, promoting the acquisition of a promigratory phenotype (Head et al., 2003; Sumida and Yamada, 2015) (**Figure 1**).

**Figure 2:**
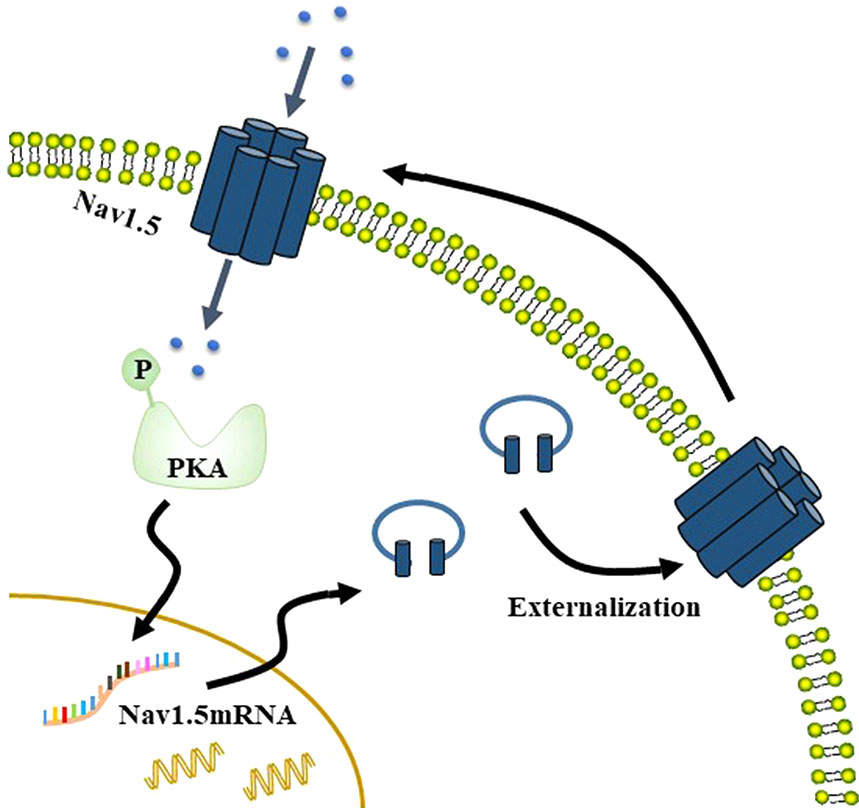
Na^+^ currents carried by Nav1.5 stimulate phosphorylation of PKA, which increases the Nav1.5 mRNA level but does not affect the total protein level. This changes the distribution of Nav1.5 in MDA-MB-231 cells, such that Nav1.5 protein level increases on the membrane but that in the cytoplasm decreases. The externalization of Nav1.5 then potentiates the invasion and migration of breast cancer cells. The increase of Nav1.5 on the membrane in turn increases the Na^+^ currents, thus, forming a positive feedback.

Na^+^ currents carried by Nav1.5 stimulate phosphorylation of protein kinase A (PKA), which increases the Nav1.5 mRNA level but does not affect the total protein level. This changes the distribution of Nav1.5 in MDA-MB-231 cells, with the level of Nav1.5 increased on the plasma membrane and reduced in cytoplasm. The externalization of Nav1.5 potentiates the invasion and migration of breast cancer cells. The increase of Nav1.5 on the membrane in turn increases the Na^+^ currents, thus, forming a positive feedback (**Figure 2**) (Chioni et al., 2010). There is also positive feedback between Nav1.5 and GTPase RhoA, which is overexpressed in many cancers, including breast cancer. Silencing of RhoA decreases the expression of Nav1.5 mRNA, as well as Nav1.5-mediated Na^+^ currents. In turn, silencing of Nav1.5 decreased the protein level of RhoA (Dulong et al., 2014), which may be a result of the reduction in calcium concentration (Rao et al., 2001). In addition, suppressing both RhoA and Nav1.5 significantly reduced the invasion of MDA-MB-231 cells (Dulong et al., 2014).

Recent research has shown that Nav1.5 promotes invasiveness by taking part in EMT, a process involved in tissue formation during embryogenesis and repair processes, and promoting cancer cell dissemination from the primary tumor (Banyard and Bielenberg, 2015). MDA-MB-231 cells present typical spindle-shaped mesenchymal morphology and multiple filopodia. Upon knocking down the expression of *SCN5A*, these cells show decreased the number of filopodia that are shorter in length. The expression level of SNAI1, an EMT-inducing transcription factor, significantly decreased in MDA-MB-231 cells treated with a specific small-hairpin RNA (shNav1.5 cells). In contrast, MCF-7 cells overexpressing Nav1.5 display an increased invasive capacity, as well as an increased expression level of SNAI1, which provides evidence for the role of SNAI1 in Nav1.5 activity-mediated invasive process of breast cancer. Indeed, loss of expression of SIK1 upregulates Nav1.5 activity and expression (Gradek et al., 2019). Hence, it is that low expression levels of SIK1 in breast cancer cells induce the activity and expression of Nav1.5, followed by high expression of SNAI1, triggering the EMT and metastasis observed in breast cancer cells.

### Inhibitors of Nav1.5

Nowadays, a variety of approaches, including specific antibodies, natural toxins, and pharmacological agents, are applied to reduce the metastasis of breast cancer cells by inhibiting Nav1.5 (**Table 1**).

**Table 1:** Inhibitors of Nav1.5.

Inhibitor	Type	Effects on cancer cells	Mechanism	References
NESOpb/E3Ab	Antibodies	Reduce migration and invasion	Specifically inhibit Na_V_1.5	Brackenbury et al. (2007) Gao et al. (2019)
Phenytoin	Antiarrhythmic and antiepileptic agent	Reduce mobility migration and invasion	Inhibit Na+ currents down-regulate EGF expression and ERK1/2 phosphorylation reduce activity of MP-7, cathepsin E and kallikrein-10	Yang et al. (2012) Mohammed et al. (2016) Nelson et al. (2015a)
DAPT	Notch signal inhibitor	Reduce proliferation lateral motility	Increase the level of Notch4mRNA and decreased the ratio of MMP9 to TIMP1	Aktas et al. (2015)
DHA	ω-3 LC-PUFA	Reduce migration and invasion	Inhibit the expression of PPARβ and downregulate SCN5A expression and activity of NHE-1	Isbilen et al. (2006) Wannous et al. (2015)
Ranolazine	Antiarrhythmic drug	Reduce invasion	Decreases Na+ influx and ECM degradative activity	Driffort et al. (2014)
Taxol	Anticancer drug	Decrease invasion	Left shift of Na_V_1.5 activation and affect microtubule polymerization	Tran et al. (2009)
FS50	Animal salivary protein	Reduce migration and invasion	Decrease the expression of Na_V_1.5 mRNA level and distribution of Na_V_1.5 protein in the cell membrane	Zhang et al. (2018)
Compounds	Synthetic substances	Reduce the invasion	Block Nav1.5 channel	Dutta et al. (2018)
Propranolol	β‐AR blocker	Reduce migration and invasion	Affect the properties and expression of Nav1.5	Lee et al. (2019)

Application of an anti-peptide polyclonal antibody, NESOpAb, blocks VGSC currents of EBNA-293 cells transfected with nNav1.5 and has no significant effect on adult Nav1.5 transfectants; thus, NESOpAb specifically targets nNav1.5, but not adult Nav1.5 (Chioni et al., 2005). NESOpAb inhibits the migration and invasion of MDA-MB-231 cells by suppressing the functional nNav1.5 activity but does not show similar results in human metastatic prostate cancer PC-3M cells, in which Nav1.7 is dominant (Diss et al., 2001; Brackenbury et al., 2007), which is consistent with the notion that NESOpAb specifically recognizes nNav1.5. E3Ab is a peptide-specific polyclonal antibody that recognizes the third extracellular region of Nav1.5, leading to specific inhibition of Nav1.5 activity (Xu et al., 2005). MDA-MB-231 cells treated with E3Ab show significantly decreased migration and invasive abilities. Indeed, a marked anti-proliferative effect was observed in E3Ab-treated MDA-MB-231 cells (Gao et al., 2019).

Phenytoin is an antiarrhythmic and antiepileptic agent that shows a high affinity toward Nav1.5 on the membrane of metastatic breast cancer cells in its inactivated state and reduces Na^+^ currents. The depolarization of MDA-MB-231 causes the opening of VGSCs, which then rapidly enter an incomplete inactivated state after a few milliseconds. This process produces transient and persistent Na^+^ currents, which play a key role in the metastasis of breast cancer cells (Yang et al., 2012). Phenytoin significantly reduces the mobility, migration, and invasion of metastatic breast cancer *in vitro* and *in vivo* (Nelson et al., 2015a; Mohammed et al., 2016). Blocking the Na^+^ current by phenytoin results in the downregulation of EGF expression and EGF-induced ERK1/2 phosphorylation, reducing the activity of MMP7, cathepsin E, and kallikrein-10, among which phenytoin showed the greatest inhibitory effect on cathepsin E (Mohammed et al., 2016). It has been proven that higher cathepsin E levels in the serum are positively correlated with poorer clinical prognosis in breast cancer patients (Kawakubo et al., 2014), thus, phenytoin might serve as an effective drug for improving therapeutic efficiency and survival of patients (Mohammed et al., 2016). Although phenytoin does not affect proliferation *in vitro* (Yang et al., 2012), it reduces the proliferation of metastatic breast cancer and slows tumor growth *in vivo* (Nelson et al., 2015a). The Notch signal inhibitor *N*-[*N*-(3,5-difluorophenacetyl)-l-alanyl]-*S*-phenylglycine-*t*-butyl ester (DAPT) has an anti-proliferative effect on MDA-MB-231 cells that can be decreased by phenytoin. Both DAPT and phenytoin reduce the lateral motility of breast cancer cells. It is reasonable to speculate that they have different effects on the proliferation of breast cancer cells. DAPT reduces the nNav1.5 mRNA expression level, which is not observed in the phenytoin treatment group. DAPT significantly reduces the ratio of MMP9 to tissue inhibitor of metalloproteinases-1 (TIMP1), which could be partly reversed by the combination of DAPT and phenytoin, indicating that the application of DAPT together with phenytoin is not a better choice than the single treatment of DAPT and phenytoin (Aktas et al., 2015). Furthermore, phenytoin inhibits the metastasis of breast cancer cells to other organs such as the lungs, liver, and spleen by reducing the density of MMP9-expressing cells (Nelson et al., 2015a). This suggests that the contribution of Nav1.5 to tumor growth *in vivo* is closely associated with adjacent tissues or the ECM.

It is noteworthy that some dietary habits have been shown to protect against cancer. It has been reported that omega-3 long-chain polyunsaturated fatty acids (ω-3 LC-PUFAs) play a role in the prevention of cancer. The effects of docosahexaenoic acid (DHA), one of the most important ω-3 LC-PUFAs, have been studied in the field of breast cancer. Short- and long-term applications of DHA inhibit nNav1.5 activity in the metastatic human MDA-MB-231 breast cancer cell line by decreasing the peak VGSC current density. In the long-term application, DHA inhibits both mRNA and protein expression levels of nNav1.5 and reduces cell migration (Isbilen et al., 2006). Peroxisome proliferator-activated receptor β (PPARβ) regulates the expression of numerous genes, including *SCN5A*, by binding with PPAR response elements on the promoters of these genes, thus, regulating cell survival, inflammation, and metabolism (Peters et al., 2012). DHA, a natural ligand of PPAR, has shown the potential to suppress tumor growth in different kinds of models with no side effects (Forman et al., 1997; Yao et al., 2014). DHA has been shown to reduce PPARβ expression, which is overexpressed in breast cancer cells, and inhibits the invasiveness and growth of breast cancer cells (Wannous et al., 2013; Yao et al., 2014; Wannous et al., 2015). The downregulation of PPARβ reduces *SCN5A* expression at both the mRNA and protein levels, as well as nNav1.5 density at the plasma membrane, which finally leads to the reduction of Na^+^ currents. In addition, the downregulation of PPARβ reduces NHE-1–dependent H^+^ efflux by inhibiting the activity of Nav1.5 channels without altering NHE-1 expression. PPARβ is indispensable for DHA to reduce Nav1.5 expression and NHE-1 activity, giving rise to the invasiveness of breast cancer cells (Wannous et al., 2015).

Ranolazine has been approved by the US Food and Drug Administration for chronic angina (Antzelevitch et al., 2004). One of the most characterized pharmacological effects of ranolazine is its ability to selectively inhibit late Na^+^ currents. The inhibition of Na^+^ currents triggers a steeper Na^+^ gradient and activation of the Na^+^/Ca exchanger, which reduces the intracellular overloaded calcium and improves ventricular relaxation in cardiac ischemia conditions (Fraser et al., 2006). Ranolazine has also been shown to have anti-invasiveness potential in breast cancer. *In vitro*, ranolazine reduces the function of Nav1.5 and decreases Na^+^ influx in MDA-MB-231 cells, which results in decreased ECM degradative activity and pro-invasive morphology of cells. *In vivo*, ranolazine slows down tumor growth and inhibits the colonization of breast cancer cells to other organs without obvious toxic effects by reducing the Nav1.5 carried currents in tumor tissues (Driffort et al., 2014). The expressions of β‐adrenergic receptor (β‐AR) and Nav1.5 overlap substantially in MDA-MB-231 cells. Propranolol which is a blocker of β‐AR, coupled to with PKA activation, modulates Nav1.5 and takes part in reducing migration and invasion of MDA-MB-231 cells. Short-term treatment with propranolol tends to reduce peak Na^+^ currents carried by Nav1.5, while long-term treatment results in sustained changes of properties and expression of Nav1.5. Indeed, the role of factors contained in the serum cannot be ignored. Both propranolol and ranolazine reduced the motility and invasiveness of MDA-MB-231 cells, but the effect of their combination was not better than that of individual treatments (Lee et al., 2019).

Taxol and its derivatives, such as docetaxel, are widely used in the treatment of breast cancer (Dang and Hudis, 2006). The widely accepted explanation for their effects is that they stabilize the microtubules during mitosis, which leads to the inhibition of cell division (Camacho et al., 2000). Taxol also has a potential anti-invasive effect on breast cancer cells at low concentrations but has no effect on cell proliferation. The reduction of the current carried by Nav1.5 was not observed in cells pretreated with taxol, which demonstrates a significant leftward shift of the activation properties of Nav1.5. It is known that the activity of sodium channels is closely associated with perturbation of microtubules (Motlagh et al., 2002). Tran reported that a short-term and low-dose taxol treatment is sufficient to affect microtubule polymerization (Tran et al., 2009). It seems that the effects of taxol on cell invasiveness are complicated, and other proteins involved in the Nav1.5-regulated signaling pathway may be regulated by taxol, but the precise mechanisms remain unknown.

Recently, animal peptides have drawn considerable interest. FS50, the salivary protein from *Xenopsylla cheopis*, shows an inhibitory effect against the Nav1.5 channel (Xu et al., 2016). FS50 significantly reduced the peak VGSC currents and decreased the expression of Nav1.5 mRNA in MDA-MB-231 cells but had no effect on the total Nav1.5 protein levels. Notably, FS50 reduces the Nav1.5 protein expression in the plasma membrane, which means that FS50 only changes the distribution of the protein between the plasma membrane and the cytoplasm. The effect is identical to the effects of a PKA inhibitor; thus, FS50 may inhibit the PKA pathway, resulting in the reduced expression of Nav1.5. In the future, it is worth detecting PKA-related proteins after the treatment of FS50 (Brackenbury and Djamgoz, 2006; Chioni et al., 2010). FS50 inhibits the migration and invasion of MDA-MB-231 cells but has no effect on the proliferation of MCF-7 and MDA-MB-231 cells. The reduction of MMP9 activity and the ratio of MMP9 mRNA to TIMP1 mRNA were observed in MDA-MB-231 cells treated with FS50 (Zhang et al., 2018).

In addition to the known VGSC-blocking drugs, scientists have developed a highly predictive and comprehensive three-dimensional quantitative structure–activity relationship model for designing the compounds to bind with VGSC ligands (Zha et al., 2014). Five low micromolar, small molecule compounds acting as Nav1.5 blockers in MDA-MB-231 cells were designed, synthesized, and evaluated. Two of the compounds were identified to reduce peak Na^+^ currents and reduce the invasion of MDA-MB-231 cells without affecting cell viability (Dutta et al., 2018). Compared with known drugs, these compounds are more effective and have simpler chemical structures. Moreover, synthetic substances are designed to target Nav1.5, providing a new and potent direction to develop drugs for the treatment of breast cancer metastasis.

The membrane current generated by VGSCs has two distinct modes: transient (I_NaT_) and persistent (I_NaP_). I_NaP_ can last 100 ms to 1 s, while I_NaT_ lasts a millisecond (Djamgoz and Onkal, 2013). Hypoxia, which is commonly observed in cancer and promotes metastasis and invasion, significantly contributes to increasing I_NaP_ (Hammarstrom and Gage, 2002; Muz et al., 2015; Rankin and Giaccia, 2016; Guzel et al., 2019). Compared with I_NaT_, I_NaP_ is resistant to inactivation even at depolarized potentials and will lead to significant changes in the global level of Na^+^ that affect Nav1.5-dependent mechanisms and play an important role in cancer progression (Saint, 2006). Indeed, the inhibition of I_NaP_ has been shown to produce a major anti-metastatic effect and has been proposed as a new target (Guzel et al., 2019). As mentioned above, MDA-MB-231 cells exhibit depolarized Vm. In addition, slower kinetics of activation, inactivation, and recovery from inactivation are observed in nNav1.5 than in the ‘adult’ form (Onkal et al., 2008). Thus, it is reasonable to assume that I_NaP_ is mainly responsible for Na^+^ elevation in breast cancer cells. At present, it is easy to identify I_NaT_ and I_NaP_ by whole-cell patch-clamp recordings, and a number of pharmacological agents have been shown to selectively block I_NaP_ (Urbani and Belluzzi, 2000; Maier, 2009). In the future, additional I_NaP_ blockers inhibiting Nav1.5-mediated persistent current should be considered as a new target to reduce metastasis of breast cancer. In addition, most agents listed above produce a major anti-metastatic effect by decreasing the current carried by Nav1.5 or changing the properties of Nav1.5. Specifically, decreasing the expression of nNav1.5 is another ideal target for specific anti-breast cancer treatment.

## Perspectives

The increasing evidence is indicative of the role of aberrant Nav1.5 activation in the metastatic progression of breast cancer cells. Nav1.5 functions to trigger a variety of downstream mechanisms in breast cancer cells to regulate metastatic and invasive capacity. Considering the large and growing body of evidence, different approaches recognizing Nav1.5 are applied to inhibit metastasis of breast cancer *in vitro* and favor Nav1.5 as an anti-metastatic target. Although we reviewed several studies here, there are many unanswered questions that require further investigation: (i) Is it possible to make nNav1.5 a biomarker of early diagnosis of breast cancer; (ii) Can we develop additional agents specifically blocking I_NaP_ or nNav1.5 to improve efficacy and reduce side effects; and (iii) How can we accumulate the process of translational medicine and promote the application of these agents in the clinic to improve the outcomes for breast cancer patients.

## Author Contributions

QL and TW wrote the review and designed the figures and the table. WW and GC made contributions to searching related articles. XL, LJ, HT, MR and SK contributed to the conception of the study. MD supervised the whole work, contributed to writing, and critically revised the paper. All authors contributed to the article and approved the submitted version.

## Funding

This work was supported by the National Natural Science Foundation of China under contract (Nos. 31672290, 31100764, 31971190, 30901874, 81503276), the Natural Science Foundation of Hunan Province, China (No. 2016JJ3180), the Valuable Instrument and Equipment Fund of Central South University (No. CSUZC2019046, CSUZC2020043).

## Conflict of Interest

The authors declare that the research was conducted in the absence of any commercial or financial relationships that could be construed as a potential conflict of interest.
